# A Cascaded DNA Nanocircuit for Multi‐Signal‐Responsive Precision siRNA Delivery in Cancer Therapy

**DOI:** 10.1002/advs.77332

**Published:** 2026-08-24

**Authors:** Yan Zhao, Yufei Lan, Min‐Goo Lee, Jinjun He, Yueran Pan, Eunji Kim, Xin Fu, Sungwook Jung, Wanxia Lu, Jinyue Fan, Haoxiang Chen, Yuanbo Zhao, Yuling Xu, Jong Seung Kim, Chao Zhang

**Affiliations:** ^1^ School of Chemical Engineering and Technology Sun Yat‐sen University Zhuhai China; ^2^ Institute of Biomedical Health Technology and Engineering Shenzhen Bay Laboratory Shenzhen China; ^3^ Department of Neurosurgery Zhujiang Hospital Southern Medical University Guangzhou China; ^4^ Department of Chemistry Korea University Seoul South Korea

**Keywords:** cancer therapy, cascaded logic gates, DNA nanocircuits, multi‐signal response, siRNA delivery

## Abstract

Precision control over nucleic acid delivery remains a critical challenge in cancer therapy, particularly for siRNA‐based gene silencing, where off‐target effects limit clinical translation. Herein, we report a programmably engineered DNA nanocircuit with cascaded dual‐AND logic gates, which enables the development of a spatiotemporally controlled siRNA delivery strategy for precision cancer therapy. The DNA nanocircuit is engineered to respond to three tumor‐specific signals in a sequential manner: extracellular acidic pH, membrane‐overexpressed nucleolin (NCL), and intracellular glutathione (GSH). The first AND gate is activated by the co‐occurrence of acidic pH and NCL, triggering a conformational rearrangement that generates a molecular output. This integrated output, combined with intracellular GSH, serves as the dual input to co‐activate the second AND gate, initiating siRNA release via a cascade reaction inherent to the DNA circuit. As a proof‐of‐concept, when harnessing this DNA circuit in a temozolomide (TMZ)‐resistant glioblastoma (GBM) mouse model, we demonstrate that this design ensures highly selective release of siPARP1 in GBM cells, achieving efficient PARP1 silencing, reversed TMZ resistance, and minimized off‐target toxicity. Collectively, the cascaded dual‐AND logic, enabled by precise DNA sequence programming, represents a generalizable strategy for multi‐signal‐responsive delivery systems, highlighting the potential of DNA circuits in precision cancer therapy.

## Graphical abstract

A cascaded dual‐AND logic DNA nanocircuit is engineered to respond to three tumor‐specific signals in a sequential manner: extracellular acidic pH, membrane‐overexpressed nucleolin (NCL), and intracellular glutathione (GSH). This programmable system selectively releases siPARP1 in glioblastoma (GBM), effectively silencing PARP1 and reversing TMZ resistance with minimal off‐target effects.

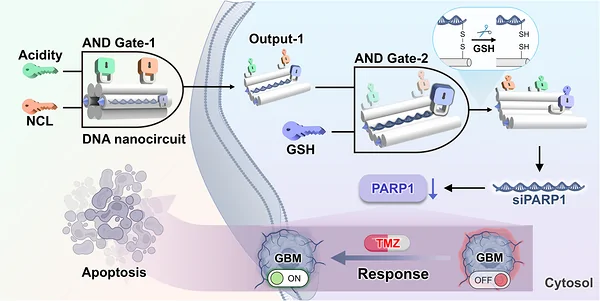

## 1 Introduction

Controlled delivery systems that selectively act on tumor cells while sparing normal tissues are pivotal for advancing siRNA‐based gene silencing therapy in cancer treatment [1, 2, 3]. However, their clinical translation is severely hampered by imprecise spatiotemporal control over cargo release. In siRNA‐based therapies, this shortcoming is especially detrimental, as off‐target gene silencing in healthy cells can disrupt essential biological processes (e.g., PARP1‐mediated DNA repair), triggering severe systemic toxicity and drastically limiting therapeutic efficacy [4, 5, 6, 7]. The root cause lies in the inability of current systems to specifically verify tumor identity, a flaw that becomes even more aggravated in complex microenvironments like glioblastoma (GBM) [8, 9]. In GBM, tumor cells share partial features with normal tissues, making accurate cargo delivery to only tumor cells a formidable challenge [10, 11].

Existing stimuli‐responsive delivery systems predominantly rely on single or non‐sequential dual signals, a design highly prone to false activation [12, 13, 14, 15, 16]. For instance, acidic pH, a hallmark of the tumor extracellular environment, also exists in inflamed tissues [11, 17, 18, 19]. Tumor‐associated markers like nucleolin (NCL) and high levels of glutathione (GSH, an antioxidant) within tumors can be minimally or modestly expressed in healthy cells [20, 21]. Systems responding to such isolated signals will inevitably misdeliver cargo to non‐tumor sites. This not only undermines therapeutic precision but also leads to off‐target siRNA silencing in healthy cells, which can disrupt normal biological functions and cause systemic toxicity [22, 23, 24].

DNA nanotechnology, with its sequence‐programmable logic operations, offers a promising solution [25, 26, 27]. DNA circuits can process multiple inputs via logic gates and transduce signals through cascaded reactions, mimicking “multi‐parameter verification” at the molecular scale [28, 29]. However, most reported DNA logic‐based delivery systems employ single logic gates [14, 30]. This restricts their ability to integrate sequential signals across extracellular and intracellular compartments, a critical requirement for distinguishing tumor cells from normal tissues, especially in complex scenarios like GBM.

**FIGURE 1:**
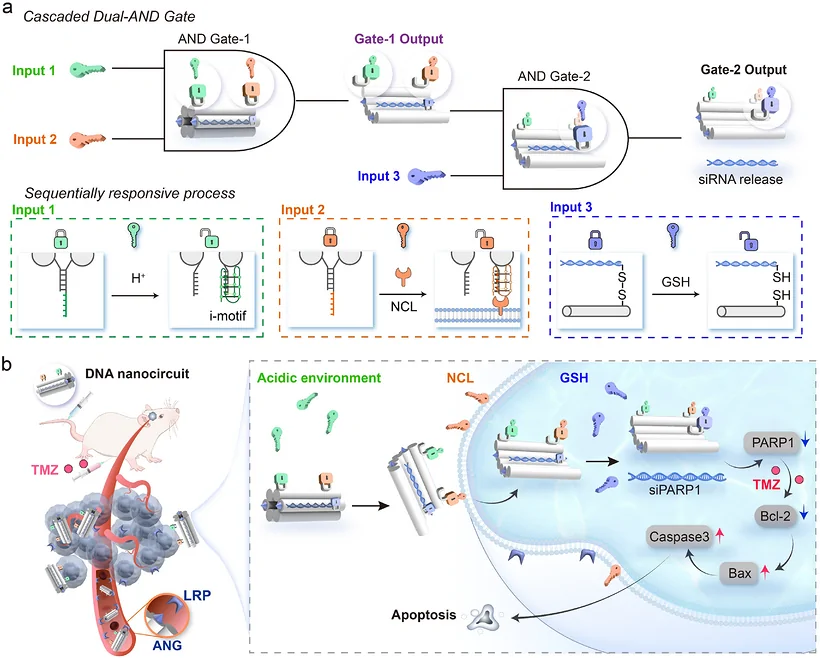
Design and therapeutic application of the cascaded dual‐AND logic DNA nanocircuit. (a) Schematic illustration of a DNA nanocircuit programmed with dual‐AND logic gates. The first AND gate responds to extracellular acidic pH (Input 1) and membrane‐overexpressed NCL (Input 2). Co‐occurrence of these two tumor‐specific signals triggers a conformational rearrangement, generating a molecular output. This output (serving as Gate‐1 output), together with intracellular GSH (Input 3), coactivates the second AND gate. (b) In vivo application in a GBM mouse model. After crossing the blood‐brain barrier (BBB) through the binding of Angiopep‐2 (ANG) to the low‐density lipoprotein receptor‐related protein (LRP, highly expressed on both brain capillary endothelial cells [31] and GBM cells), the nanocircuit responds to the sequential tumor‐specific signals (acidic pH, NCL, GSH). This enables precise siPARP1 release and gene silencing, thereby overcoming TMZ resistance and inducing significant tumor cell apoptosis.

In this study, we present a chemically engineered DNA nanocircuit featuring cascaded dual‐AND logic gates, designed to achieve stringent tumor‐specific siRNA release. The circuit is programmed to respond to three tumor‐specific signals in a strict spatiotemporal sequence: (1) extracellular acidic pH (pH∼6.5) and (2) membrane‐overexpressed NCL first activate the first AND gate, triggering a conformational rearrangement that primes the system for intracellular processing. Following cell endocytosis, this primed state acts as one input for the second AND gate, with (3) intracellular GSH (enriched in tumor cytosol) serving as the other. Only upon sequential verification of all three signals does the circuit release encapsulated siRNA (Figure 1a). This design eliminates false activation in normal tissues with isolated signals, a critical advantage over single‐gate systems. As a proof‐of‐concept, in temozolomide (TMZ)‐resistant GBM models, this circuit ensures selective siPARP1 release exclusively in GBM cells, thereby achieving efficient PARP1 silencing, reversed TMZ resistance, and minimal off‐target toxicity in normal tissues (Figure 1b), thus validating its specificity and therapeutic potential. This precision stems from rational sequence engineering (pH‐sensitive i‐motif, NCL aptamer, GSH‐cleavable linkage), and its modular design enables adaptation to diverse tumors, establishing a framework for multi‐compartment‐responsive precision delivery.

## 2 Results and Discussion

### 2.1 Design and Operation of the Cascaded Dual‐AND Logic DNA Nanocircuit

This cascaded dual‐AND logic DNA nanocircuit was constructed based on a six‐helix bundle (6HB) DNA framework [32]. On the inner side of the 6HB DNA framework, siPARP1 is locked to the framework via a disulfide bond responsive to intracellular GSH. Along with the long side of the framework, two duplex locks are used to maintain its closed conformation: one is formed between a C‐rich strand and its partially mismatched complement, and the other between an NCL‐binding aptamer and a mismatched strand (Figure S1 and Table S1).

This nanocircuit was assembled by thermal annealing of all component DNA strands and the siPARP1‐conjugated oligonucleotide, in a one‐pot reaction (Table S2). The resulting nanocircuit exhibited a uniform tubular morphology, as confirmed by atomic force microscopy (AFM) (Figure S2). The incorporation of siPARP1 and ANG was verified by native polyacrylamide gel electrophoresis (PAGE) fluorescent imaging (Figure S3). Enhanced cellular uptake mediated by ANG was further validated by confocal imaging (Figure S4).

**FIGURE 2:**
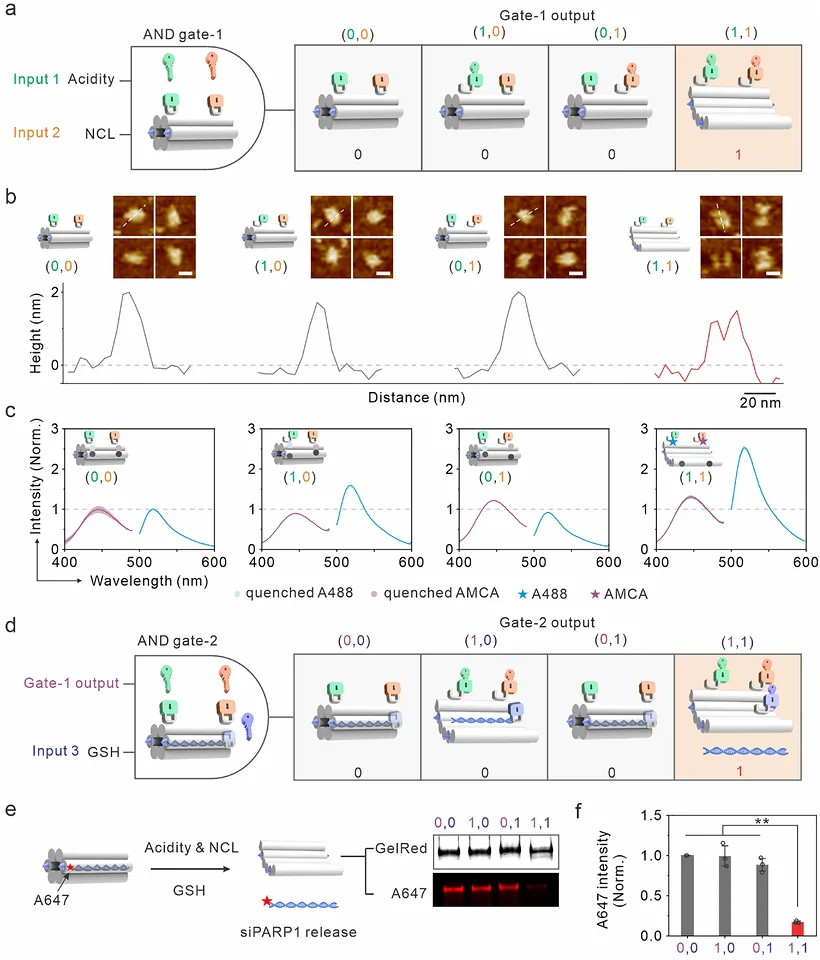
In vitro validation of cascaded dual‐AND logic gate activation. (a) The first logic gate under four different input conditions: (0,0), (0,1), (1,0), and (1,1), corresponding to the absence or presence of acidic pH (Input 1) and NCL (Input 2). The output signal is “1” only when both inputs are present. (b) AFM imaging of nanocircuit morphology under the four conditions (scale bars, 20 nm; full field in Figure S2). (c) The fluorescence patterns of the DNA nanocircuit. AMCA (*λ*_ex_ = 345 nm, *λ*_em_ = 445 nm); A488 (*λ*_ex_ = 480 nm, *λ*_em_ = 520 nm). The fluorescence intensities are normalized to the input of (0,0). Data are presented as mean ± SEM, represented by solid lines with shaded areas (n = 3). (d) Schematic of the second logic gate, activated by Gate‐1 output and GSH (Input 3), resulting in siPARP1 release. (e) Schematic of A647‐labeled siPARP1 releasing from DNA nanocircuit and PAGE analysis under four input conditions. (f) Quantification of A647 fluorescence intensity from the nanocircuit band. A647 intensities are normalized to the input of (0,0). All data are shown as mean ± SEM (n = 3), ^**^ *p* < 0.01.

To validate the stepwise responsiveness of the DNA nanocircuit, we first examined the output states of the first logic gate under four distinct input conditions: (0,0), (0,1), (1,0), and (1,1), corresponding to the absence or presence of acidic pH (Input 1) and NCL (Input 2), respectively. As shown in Figure 2a, the readout remained “0” under single‐input or no‐input conditions and switched to “1” only when both inputs were present, consistent with Boolean AND logic behavior. AFM imaging confirmed the structural transition associated with logic output (Figure 2b, full field of view shown in Figure S2). Under conditions (0,0), (1,0), and (0,1), the DNA nanocircuit maintained a closed tubular morphology with heights of 2.13, 2.06, and 2.10 nm and widths of 18.09, 18.92, and 18.63 nm, respectively. In contrast, under the (1,1) condition, the DNA nanocircuit exhibited a distinct two‐peak height profile with reduced peak values (1.51 nm) and increased width (32.94 nm), indicating partial opening of the tubular structure along the locked edge. We also employed fluorescence spectroscopy to monitor the structural transition by incorporating fluorophore‐quencher pairs into both duplex locks: Alexa488‐BHQ1 and AMCA‐Dabcyl (Figure S5).

In the closed state, fluorophores are close to their corresponding quenchers, resulting in minimal fluorescence. Upon input‐triggered unlocking, strand displacement increases the fluorophore‐quencher distance, leading to fluorescence recovery. As shown in Figure 2c and Figure S6, statistical increases in both A488 and AMCA signals were observed exclusively under the (1,1) condition, confirming that dual input is required to induce full structural opening. Time‐course measurements revealed that fluorescence reached a plateau approximately 20 min after simultaneous addition of the two inputs, indicating the kinetics of the unlocking process (Figure S7). These results validate that the first logic gate can accurately process combinatorial extracellular cues and trigger structural changes in response to dual input activation.

The second logic gate integrates the structural output of the first gate with intracellular GSH as inputs (Figure 2d). Only when both inputs are present (the nanocircuit is unlocked and GSH is abundant), can the disulfide linkage be cleaved, leading to siPARP1 release. To monitor this process, Alexa 647 (A647) was conjugated to the antisense strand of siPARP1. Native PAGE was used to assess the release behavior, with the DNA nanocircuit visualized by GelRed and siPARP1 tracked via A647 fluorescence. As shown in Figure 2e,f, under the (1,1) condition with 5 mm GSH, the A647 fluorescence intensity on the nanocircuit band significantly decreased, indicating the effective release of siPARP1 from the nanocircuit. Similar results were observed under 10 mm GSH conditions (Figure S8). In all other tested conditions, the A647 fluorescence intensity remained within the DNA nanocircuit, confirming the logic‐gated release specificity.

### 2.2 Highly Specific Cascaded Logic Control of siPARP1 Release in U87MG‐TR Cells

**FIGURE 3:**
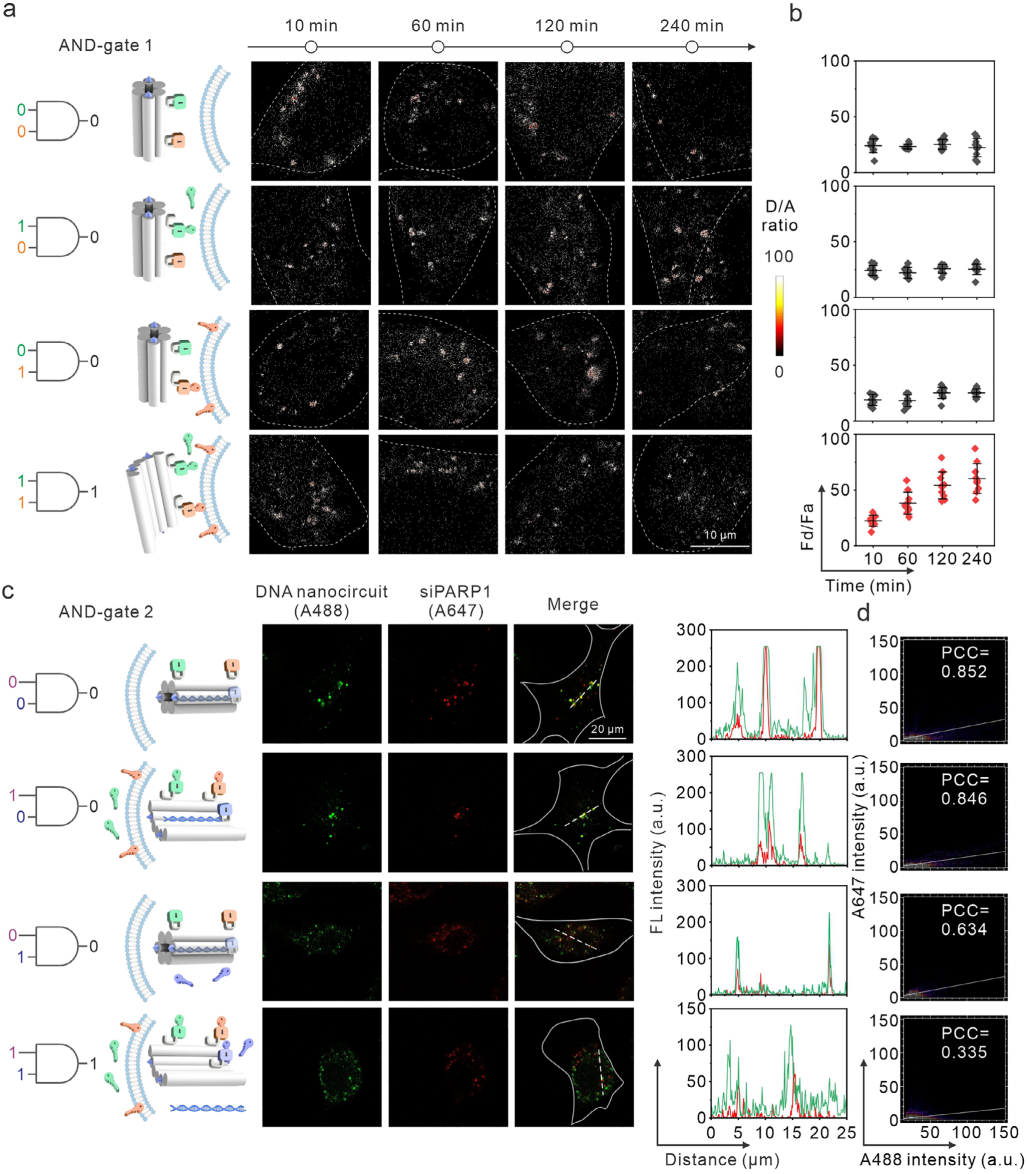
Validation of the sequential logic behavior of DNA nanocircuit in living cells. (a) Pseudocolor donor/acceptor (D/A) images depicting the temporal response of AND gate‐1 at the cell surface under different logic‐gate inputs. (b) D/A ratio quantification from panel a, based on n = 10 fluorescent puncta per time point, shown as mean ± SEM. (c) Colocalization of the DNA nanocircuit (A488‐labeled, green) and siPARP1 (A647‐labeled, red) in AND gate‐2. Line profiles show green and red fluorescence intensities along the dashed lines in the merged images. (d) Colocalization maps of the cells in panel c with the corresponding Pearson's correlation coefficients.

Before testing the sequential logic behavior of the DNA nanocircuit in living cells, we first confirmed the stability of the DNA nanocircuit and its encapsulated siPARP1 under physiological conditions (Figure S9). We then implemented AND gate‐1 on the surface of U87MG‐TR cells, using pH (acidic (∼6.5) = 1, neutral (∼7.3) = 0) and NCL expression (normal = 1, inhibited = 0) as inputs. The acidic environment was generated by pH regulation, while NCL expression inhibition was induced by serum starvation (Figure S10). Only under the dual‐input condition (acidic, normal), the Cy3/Cy5 FRET pair separated, producing a pronounced time‐dependent increase in donor/acceptor (D/A) ratio. In contrast, single or no input kept the fluorophores in proximity, yielding consistently low ratios, thus confirming logic‐dependent activation (Figure 3a,b, Figure S11).

Building on this, AND gate‐2 was designed to control siPARP1 release inside U87MG‐TR cells. Here, the inputs were the status of AND gate‐1 (open = 1, closed = 0) and intracellular GSH level (normal = 1, inhibited = 0). GSH content inhibition was achieved using the GSH synthesis inhibitor‐L‐buthionine‐(S, R)‐sulfoximine (BSO) (Figure S12) [33]. The dual‐input (1,1) condition led to siPARP1 dissociation from the nanocircuit, reflected by weak green‐red overlap and a low Pearson's correlation coefficient. Conversely, single or no input preserved strong colocalization, indicating siPARP1 sequestration without release (Figure 3c,d). On the other hand, the DNA nanocircuit remained largely inactive in normal human astrocytes (NHA) cells (Figure S13). Together, these results demonstrate that the DNA nanocircuit operates as a cascaded AND logic system in U87MG‐TR cells, integrating extracellular and intracellular cues to achieve highly specific and sequential control over siPARP1 release.

### 2.3 Selectivity and Safety of the DNA Nanocircuit in Normal Cells and Tissues

**FIGURE 4:**
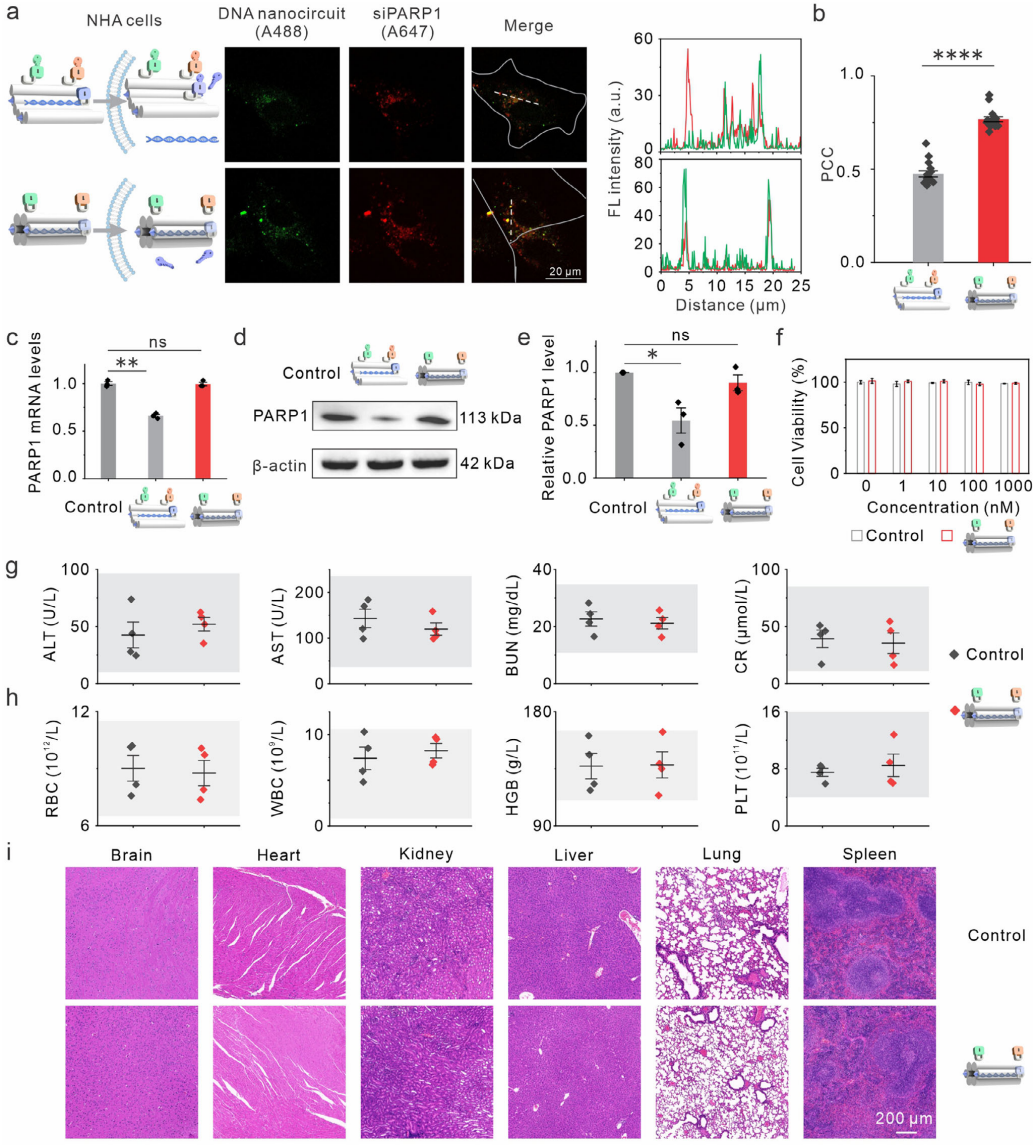
Non‐responsiveness and safety of the DNA nanocircuit in normal biological systems. (a) Colocalization of the DNA nanocircuit (A488‐labeled, green) and siPARP1 (A647‐labeled, red) in NHA cells containing AND gate‐1‐deficient DNA nanocircuit (top) or the cascaded dual‐AND logic DNA nanocircuit (bottom). Line profiles show green and red fluorescence intensities along the dashed lines in the merged images. (b) Pearson's correlation coefficients from 15 cells per condition. (c) Relative PARP1 mRNA levels in two siPARP1 release conditions determined by qPCR (n = 3). (d) Western blot (WB) analysis of PARP1 protein expression. (e) Densitometric quantification of panel d (n = 3). (f) Viability of NHA cells treated with the DNA nanocircuit at varying concentrations. (g, h) Serum biochemistry (ALT, AST, BUN, CR) and hematological parameters (RBC, WBC, HGB, PLT) in mice after injection of the DNA nanocircuit, grey areas indicate standard reference ranges (n = 4). (i) Representative hematoxylin and eosin (H&E) staining of brain, heart, kidney, liver, lung, and spleen tissues from mice post‐injection of the DNA nanocircuit. All data are shown as mean ± SEM. ns, no significant; ^*^ *p* < 0.05, ^**^ *p* < 0.01, ^****^ *p* < 0.0001.

While GSH levels in normal tissues are typically only 20%–50% of those in tumors [34], they remain non‐negligible given their critical role in scavenging physiological reactive oxygen species and mediating oxidative damage repair [35]. This substantial baseline concentration significantly increases the risk of potential unintended activation and erroneous PARP1 gene knockouts in normal cells when relying solely on a GSH‐responsive switch. To validate the necessity of our outer AND gate‐1 design, we employed an AND gate‐1‐deficient DNA nanocircuit as a control. This experimental setup enabled us to assess both the non‐responsiveness and safety profiles of our custom‐engineered cascaded dual‐AND logic DNA nanocircuit in NHA cells and normal tissues. Imaging analysis showed that siPARP1 largely remained colocalized with the cascaded dual‐AND logic DNA nanocircuit, following with a high Pearson's correlation coefficient, which indicated minimal release compared with the AND gate‐1‐deficient control (Figure 4a,b and Figure S14). Consistently, PARP1 in both mRNA and protein levels didn't exhibit obvious change in the DNA nanocircuit treatment group (Figure 4c–e), confirming that the nanocircuit does not trigger unintended gene silencing in normal cells. Moreover, cell viability assays revealed negligible cytotoxicity after the DNA nanocircuit treatment at varying concentrations (Figure 4f). In vivo, mice injected with the DNA nanocircuit exhibited stable serum biochemical and hematological parameters within normal reference ranges (Figure 4g,h and Figure S15), and histological analysis showed no tissue damage in major organs (Figure 4i, Figure S16). Taken together, these results indicate that the DNA nanocircuit is biologically inert in non‐target cells and well tolerated in vivo, highlighting its potential for selective therapeutic delivery. In addition, cytokine profiling showed no significant changes, further indicating minimal systemic immune activation (Figure S17).

### 2.4 Logic‐Gated DNA Nanocircuit for PARP1 Knockdown

**FIGURE 5:**
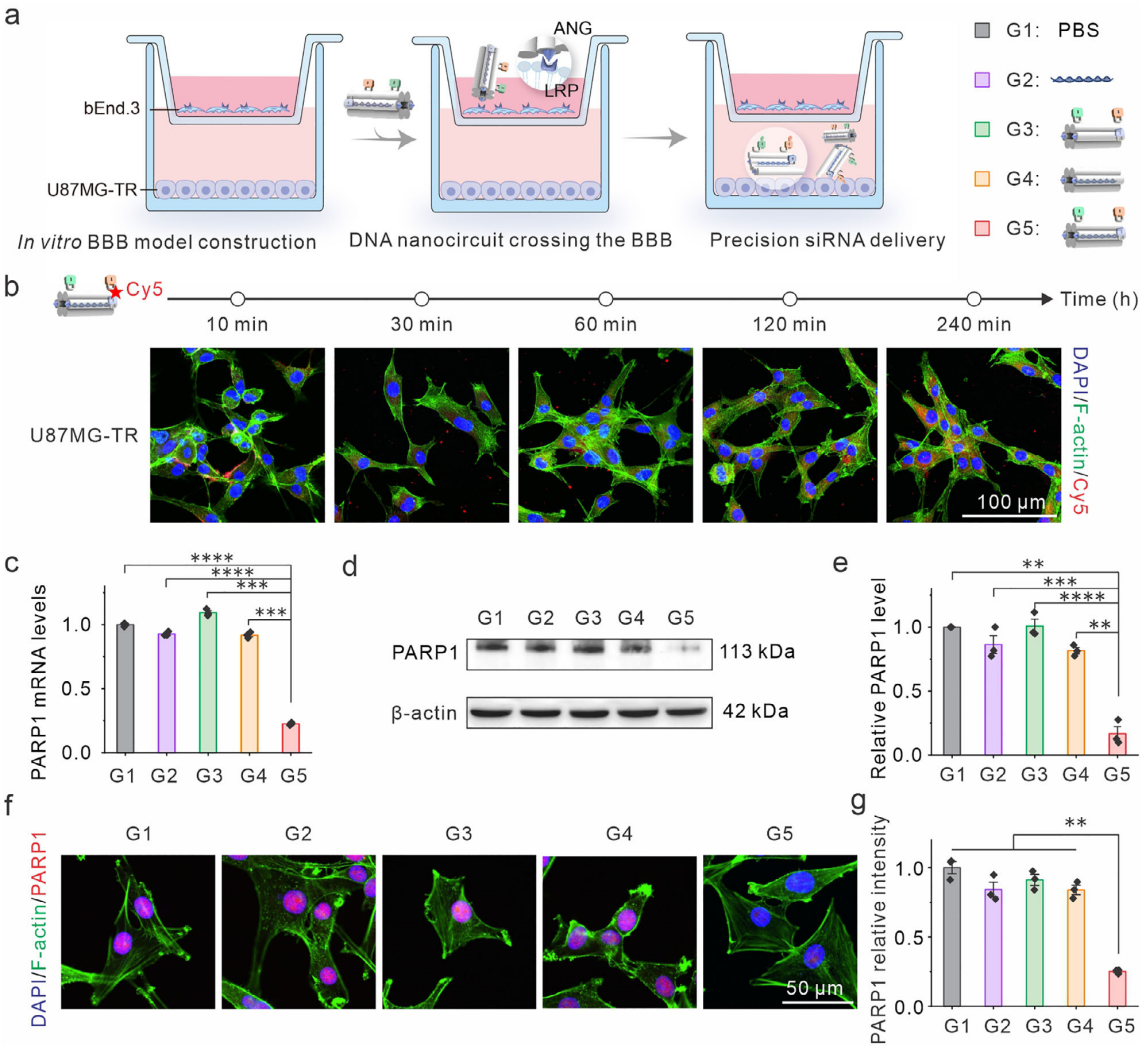
In vitro evaluation of the targeted PARP1 gene silencing effect by the DNA nanocircuit. (a) Schematic illustration of the BBB Transwell system with bEnd.3 cells cultured in the upper chamber and U87MG‐TR cells in the lower chamber. The DNA nanocircuit traverses the upper barrier via ANG and activates in the lower chamber to release siPARP1. (b) Confocal imaging of Cy5‐labeled DNA nanocircuit being uptake by lower chamber cells at the indicated time. (c) qPCR quantification of PARP1 mRNA expression under different treatments (n = 3). (d, e) WB detection of PARP1 protein (d) and corresponding densitometric analysis (e) (n = 3). (f) IF imaging of PARP1 expression in U87MG‐TR cells and (g) quantitative analysis following treatment. All data are shown as mean ± SEM, ^**^ *p* < 0.01, ^***^ *p* < 0.001, ^****^ *p* < 0.0001.

A robust capacity to traverse the blood‐brain barrier (BBB) is a fundamental prerequisite for the DNA nanocircuit to achieve effective targeted PARP1 gene silencing. To evaluate the DNA nanocircuit's ability to cross the BBB, we established a BBB Transwell model with bEnd.3 cells in the upper chamber and U87MG‐TR cells in the lower chamber as previously reported method [36, 37]. The DNA nanocircuit, guided by the ANG, successfully traversed the upper cell layer and invoked a cascaded response for siPARP1 activation in the lower chamber (Figure 5a,b and Figure S18).

Subsequently, we assessed the targeted PARP1 gene silencing efficacy of the DNA nanocircuit (Group 5: G5) by comparing it with PBS (G1), siPARP1 (G2), and function‐loss groups (G3: DNA nanocircuit without siPARP1 loaded; G4: 6HB nanostructure loaded with siPARP1 without response capability) (Figure 5c). At the protein level, WB analysis confirmed that significant PARP1 downregulation was observed exclusively in G5, highlighting the necessity of both effective delivery and tumor‐specific activation for functional gene silencing (Figure 5d,e). Immunofluorescence (IF) imaging further confirmed the reduced PARP1 expression in U87MG‐TR cells treated with the DNA nanocircuit, with preserved morphology of the nucleus and cytoskeleton (Figure 5f,g). Collectively, these results demonstrate that the DNA nanocircuit effectively suppresses PARP1 gene expression in the blood‐brain barrier model, thereby laying the foundation for reversing TMZ resistance.

### 2.5 DNA Nanocircuit Enhances TMZ Therapy in Resistant GBM Cells

**FIGURE 6:**
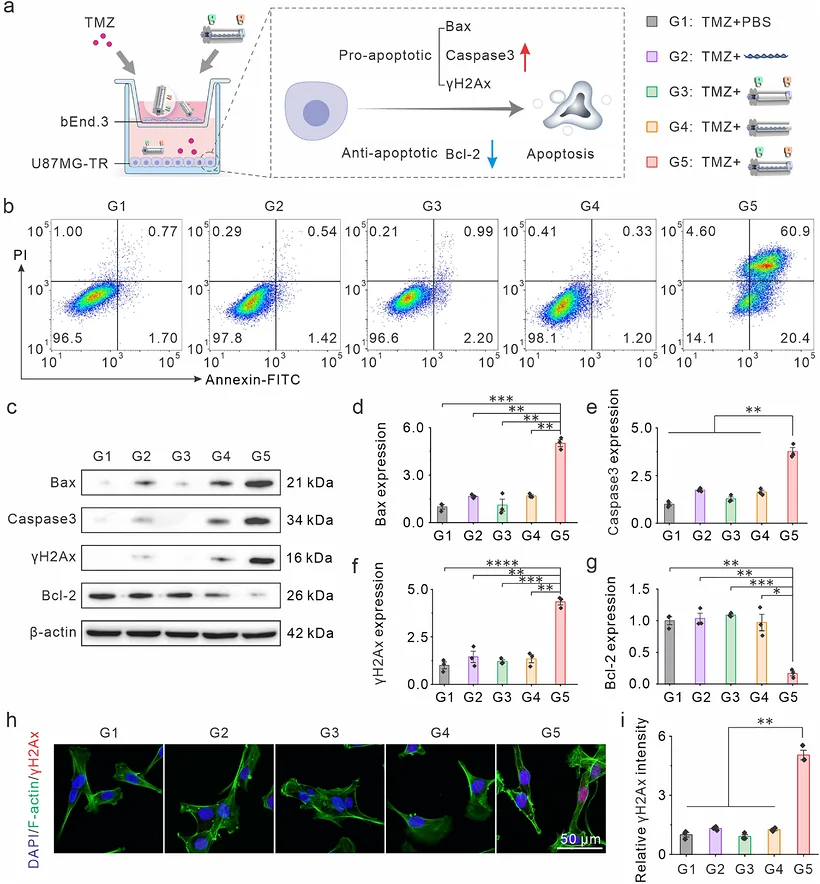
DNA nanocircuit enhances TMZ‐induced apoptosis in TMZ‐resistant GBM cells. (a) Schematic illustration of the DNA nanocircuit sensitizes TMZ‐resistant GBM cells to apoptosis through PARP1 silencing in vitro BBB model. (b) Flow cytometric evaluation of apoptosis in U87MG‐TR cells across groups. (c) WB imaging of the pro‐apoptotic and anti‐apoptotic protein expression. (d–g) Quantification of protein expression (n = 3). (h) IF staining of γH2Ax in U87MG‐TR cells following various treatments. (i) Quantification of relative γH2Ax fluorescence intensity.Data are presented as mean ± SEM, ^*^ *p* < 0.05, ^**^ *p* < 0.01, ^***^ *p* < 0.001, ^****^ *p* < 0.0001.

We then validated the efficacy of the DNA nanocircuit in killing TMZ‐resistant GBM cells when used in combination with TMZ (Figure 6a). Flow cytometry analysis indicated that the TMZ + DNA nanocircuit treatment group exhibited a significantly higher rate of apoptosis compared to the other groups (Figure 6b). While no apparent apoptosis enhancement was observed in normal NHA cells, further supporting the selective activation of the DNA nanocircuit (Figure S19). Cell Counting Kit‐8 (CCK‐8) experiments also showed that the TMZ + DNA nanocircuit treatment group had the lowest U87MG‐TR cell viability (Figure S20) and achieved the lowest half‐maximal inhibitory concentration (IC_50_) of TMZ (Figure S21).

Mechanistically, WB (Figure 6c–g) and IF analyses (Figure 6h,i) in G5 revealed pronounced upregulation of the pro‐apoptotic proteins Bax and Caspase3, accompanied by a significant increase in γH2Ax levels, indicative of enhanced DNA damage, while the anti‐apoptotic protein Bcl‐2 was markedly downregulated. Collectively, these results demonstrate that the DNA nanocircuit can effectively enhance TMZ‐induced apoptosis.

### 2.6 In Vivo Reversal of TMZ Resistance by the siPARP1‐Loading DNA Nanocircuit in Orthotopic GBM

**FIGURE 7:**
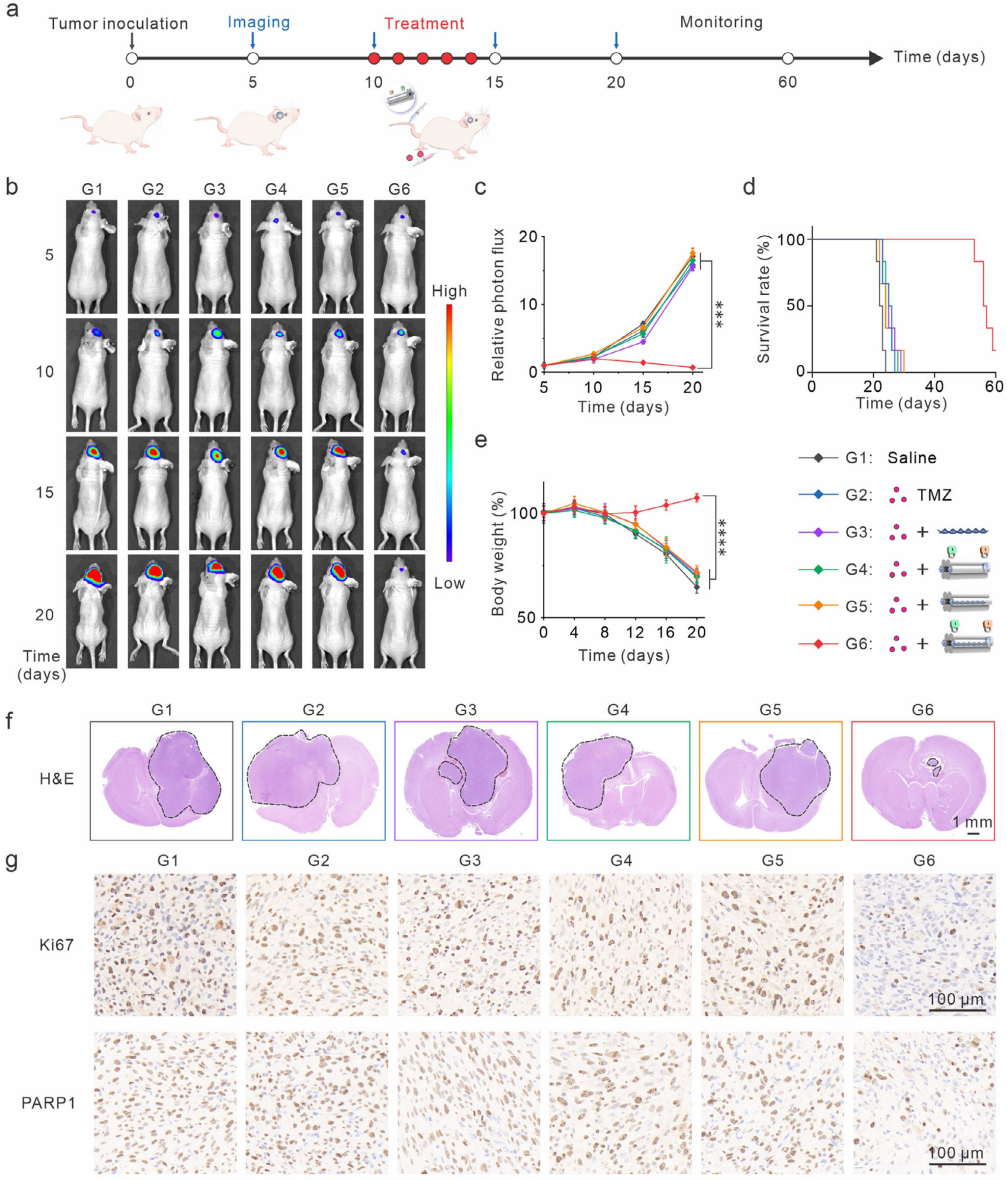
In vivo efficacy of the DNA nanocircuit in reversing TMZ resistance. (a) Schematic of the treatment and monitoring schedule in an orthotopic GBM mouse model (n = 6 per group). (b) Representative tumor bioluminescence images on days 5, 10, 15, and 20 post‐implantations. (c) Quantitative analysis of bioluminescence signals from panel b. (d) Kaplan‐Meier survival plots of mice across treatment groups. (e) Variations in body weight recorded from implantation until day 20. (f) H&E‐stained brain sections, with tumor regions highlighted by black dashed lines. (g) Immunohistochemical detection of Ki67 and PARP1 in each group. All data are shown as mean ± SEM. ^***^ *p* < 0.001, ^****^ *p* < 0.0001.

To determine whether the DNA nanocircuit could overcome TMZ resistance in GBM, we established an orthotopic GBM mouse model with luciferase‐transfected U87MG‐TR cells [38]. In vivo biodistribution analysis confirmed that ANG modification facilitated preferential accumulation of the DNA nanocircuit at the tumor site (Figure S22). Mice were randomized into six treatment groups (G1: PBS; G2: TMZ; G3: TMZ + siPARP1; G4: TMZ + DNA nanocircuit without siPARP1 loaded; G5: TMZ + 6HB nanostructure loaded with siPARP1 without response capability; G6: TMZ + DNA nanocircuit) as indicated in Figure 6 (n = 6 per group) and received daily injections for five consecutive days (Figure 7a). Serial bioluminescence imaging revealed rapid tumor growth in all the G1‐G5 groups, whereas mice treated with TMZ + DNA nanocircuit showed markedly slower tumor progression, with significantly reduced signal intensity from day 10 onward (Figure 7b,c), indicating effective delivery and targeted inhibition of siPARP1. Consistent with these observations, Kaplan‐Meier analysis demonstrated a significant survival advantage in the TMZ + DNA nanocircuit group compared with G1‐G5 groups (Figure 7d). Body weight in mice treated with the TMZ + DNA nanocircuit showed a slight increase, indicating effective therapy without apparent adverse effects (Figure 7e). Histological analysis confirmed reduced tumor burden, as smaller tumor regions were evident in H&E‐stained sections (Figure 7f), while immunohistochemistry (IHC) revealed decreased Ki67‐positive proliferating cells and suppressed PARP1 expression (Figure 7g), corroborating functional gene silence in vivo. Further analyses revealed upregulation of pro‐apoptotic proteins Bax, Caspase3, and γH2Ax, together with downregulation of the anti‐apoptotic protein Bcl‐2 (Figure S23), and increased apoptotic cells detected by Terminal Deoxynucleotidyl Transferase‐Mediated dUTP Nick End Labeling (TUNEL) staining (Figure S24), collectively supporting enhanced tumor cell death in the TMZ + DNA nanocircuit‐treated group. Together, these results demonstrate that the DNA nanocircuit enables targeted siPARP1 release within the brain, effectively reverses TMZ resistance, and confers a pronounced therapeutic benefit in an orthotopic GBM model.

## 3 Conclusions

In conclusion, this study introduces a cascaded dual‐AND logic DNA nanocircuit designed for precise siRNA delivery responsive to multiple signals, tackling the enduring issue of spatiotemporal regulation in nucleic acid‐mediated cancer treatment. Through hierarchical activation of dual‐AND logic gates, this design fundamentally addresses the constraints of traditional single‐ or non‐sequential dual‐signal systems, thereby reducing off‐target toxicity. Compared to clinically established siRNA delivery platforms such as lipid nanoparticles and GalNAc conjugates, which are primarily optimized for hepatic targets and lack tumor‐specific activation mechanisms in the central nervous system, the present nanocircuit offers a programmable and logic‐gated alternative with demonstrated BBB penetration via ANG‐mediated LRP1‐dependent transport. In proof‐of‐concept studies utilizing a TMZ‐resistant GBM mouse model, the nanocircuit specifically released siPARP1 in GBM cells, reversed TMZ resistance, and diminished systemic toxicity. Safety evaluations in normal astrocytes and murine tissues further validated non‐responsiveness and tolerability in non‐targeted systems. The cascaded dual‐AND logic DNA nanocircuit facilitates the progression of siRNA‐based therapies toward clinical application, presenting a promising pathway for targeted cancer treatment.

Looking ahead, the signal‐responsive elements employed here are each independently replaceable modules within the sequence‐programmable DNA nanotechnology, enabling in principle, the adaptation of this architecture to alternative tumor microenvironmental signals or therapeutic payloads beyond the GBM context explored in this study. From an applied perspective, the exceptional stability of DNA molecules and recent advances in scalable enzymatic synthesis provide a practical foundation for the industrial translation of DNA nanocircuit‐based therapeutics. Collectively, this work establishes a generalizable and programmable framework for precision nucleic acid delivery, with broad implications for next‐generation cancer therapeutics.

## 4 Experimental Section

### 4.1 Materials

DNA oligonucleotides, siPARP1, and peptide nucleic acid (all HPLC purified) were purchased from Sangon Biotech (Shanghai, China), with sequences provided in Table S1. GSH was also obtained from Sangon Biotech. The GSH assay kit and CCK‐8 kit were purchased from Dojindo. Antibodies for Western blot analysis were sourced from Abcam and Proteintech.

The NHA, bEnd.3, and U87MG cell lines were purchased from the Cell Bank of the Chinese Academy of Sciences (Shanghai, China). U87MG‐TR (TMZ‐resistant) cells were constructed based on our previous research [39]. All cells were cultured in DMEM medium supplemented with 10% FBS and 1% penicillin/streptomycin in a humidified 37°C and 5% CO_2_ incubator.

Female BALB/c mice (6–8 weeks old) were purchased from Guangdong Medical Laboratory Animal Center and used under the animal experiment protocols approved by the Central Laboratory of Zhujiang Hospital, Southern Medical University (LAEC‐2024‐264).

### 4.2 DNA Nanocircuit Construction

First, a one‐step synthesis method was used to prepare a DNA nanocircuit without targeted peptide modification. The DNA nanocircuit was prepared by first mixing the strands listed in Table S2 at equimolar concentrations (1 µm) in 100 µL of synthesis buffer (40 mm Tris, 20 mm acetic acid, 2 mm EDTA, and 50 mm magnesium acetate, with pH adjusted to 8.0). The mixture was then slowly cooled from 95°C to room temperature in a water bath overnight. After cooling, a tenfold excess of peptide nucleic acid was added, and the resulting solution was incubated at 37°C for 10 min to yield the final DNA nanocircuit.

### 4.3 AFM Imaging

DNA nanocircuits (10 nm, 10 µL) were loaded onto mica surfaces pretreated with aminopropyltriethoxysilane and allowed to absorb for 5 min. Subsequently, the samples were scanned in liquid using a Fluid+ probe (Veeco) and detected using Bruker AFM.

### 4.4 Fluorescence Analysis of AND‐Gate 1 Response

The initial DNA nanocircuit contained two fluorophore‐quencher pairs, namely Alexa 488‐BHQ1 and AMCA‐Dabcyl, positioned at the pH and NCL‐responsive locks, respectively (Figure S5). For each test, 100 µL of 300 nM DNA nanocircuit in synthesis buffer was incubated at room temperature for 30 min under one of four conditions: no inputs, acidic (pH 6.5, adjusted with HCl) without NCL, neutral with NCL, or acidic with NCL. The molar ratio of NCL to the DNA nanocircuit was 4:1. After incubation, each mixture was transferred to a 96‐well black assay plate. Fluorescence measurements were carried out on a microplate reader, with excitation and emission settings of 480/520 nm for Alexa 488 and 345/445 nm for AMCA. Kinetic fluorescence changes during nanocircuit operation were recorded every 30 s.

### 4.5 Native Gel Electrophoresis

DNA nanocircuits after annealing were run on native PAGE gel (6% acrylamide gel) to verify their formation (Figure S3). The electrophoresis was carried out on a Bio‐Red gel electrophoresis system under 100 V for 1.5 h at room temperature in TAE Mg buffer (40 mm Tris‐HCl, 2 mM EDTA, 20 mm acetic acid, and 12.5 mm Mg Acetate). After electrophoresis, the gel was stained with GelRed (Biotium, USA). For AND‐gate 2 verification, four kinds of inputs (no inputs, acidic/NCL and no GSH, neutral/no NCL and 5 mm GSH, acidic/NCL and 5 mm GSH) were added to the DNA nanocircuit and incubated at 37°C for 12 h. Imaging was conducted by monitoring the GelRed and Alexa647 channels of the gel imaging system (Bio‐Rad).

### 4.6 Confocal Imaging and Analysis

To assess FRET, donor (Cy3) and acceptor (Cy5) fluorescence images were captured at four time points (10, 60, 120, and 240 min) following treatment of the DNA nanocircuit with different inputs. Donor signals were recorded with 561 nm excitation and 580–620 nm emission, while acceptor signals used 561 nm excitation and 650–700 nm emission. The FRET efficiency per spot was calculated as the donor/acceptor fluorescence intensity ratio using ImageJ. For colocalization analysis, A488 and A647 channel images were taken 4 h post‐input treatment, and the Pearson correlation coefficient (PCC) was determined with the JACoP plugin in ImageJ.

### 4.7 Protein and RNA Detection

For WB analysis, protein samples were extracted on ice from cultured cells and mouse GBM tissues using a membrane protein extraction kit (KeyGEN BioTECH) and a whole‐cell lysis kit (KeyGEN BioTECH). Protein concentration was then determined with a BCA assay kit (Beyotime). The extracted proteins were separated via SDS‐PAGE, incubated overnight with the primary antibody, and subsequently treated with horseradish peroxidase (HRP)‐conjugated secondary antibody for 1 h. Protein bands were detected with an ECL chemiluminescence system (ImageQuant LAS500, General Electric), and ImageJ software was used for quantification, with β‐actin serving as the internal reference. The antibody information is listed in Table S3.

For qRT‐PCR experiments, total RNA was isolated from GBM cells using the SteadyPure Rapid RNA Extraction Kit (AccurateBiology). Reverse transcription was performed with the Prime ScriptTM RT Kit (Takara Bio) according to the manufacturer's instructions, generating cDNA templates. Quantitative PCR was carried out using SYBR Green PCR Master Mix (Takara Bio) on an ABI StepOne Plus real‐time instrument (Thermo Fisher Scientific). ACTB was used as a housekeeping gene for normalization, and data were analyzed with the 2^−ΔΔCt^ method based on at least three independent replicates. The primer sequences are listed in Table S4.

### 4.8 Biosafety Experiment

Collect peripheral blood from mice. After allowing the blood sample to stand at room temperature for 30 min, centrifuge at 3000 r/min for 15 min to separate the upper serum for blood biochemistry testing and the lower blood cells for blood routine testing. Use a fully automated blood analyzer to detect blood routine indicators, including red blood cell count (RBC), white blood cell count (WBC), hemoglobin content (HGB), and platelet count (PLT); Using a fully automated biochemical analyzer to detect serum biochemical indicators, including alanine aminotransferase (ALT), aspartate aminotransferase (AST), blood urea nitrogen (BUN), and blood creatinine (CR). All testing operations strictly follow the instrument instructions.

Collect peripheral blood from mice, let the blood sample stand at room temperature, centrifuge (3000 r/min, 15 min), and separate the serum. Use enzyme‐linked immunosorbent assay (ELISA) to detect the levels of inflammatory factors in the serum, including tumor necrosis factor‐α (TNF‐α), interleukin‐1β (IL‐1β), interleukin‐6 (IL‐6), and interleukin‐10 (IL‐10). The experimental operation strictly follows the instructions of the ELISA kit: add the standard and the serum sample to the enzyme‐linked immunosorbent assay (ELISA) plate, incubate at 37°C for 2 h; add biotinylated antibodies after washing the plate, incubate at 37°C for 1 h; wash the plate again, add the enzyme conjugate, and incubate at room temperature in the dark for 30 min; After washing the plate, add the substrate color solution and incubate in the dark for 15 min. After terminating the reaction, read the absorbance values of each well at a wavelength of 450 nm. Draw a standard curve based on the concentration of the standard substance and calculate the concentration of various inflammatory factors in the sample.

Mice were euthanized, and major organs including the brain, heart, liver, spleen, lung, and kidney were harvested immediately. The tissues were fixed in 4% paraformaldehyde for 24 h at room temperature, followed by routine dehydration, clearing, and paraffin embedding. Serial sections with a thickness of 5 µm were prepared and dewaxed in xylene, then rehydrated through a graded ethanol series into distilled water. The sections were stained with hematoxylin for nuclear staining, differentiated with 1% hydrochloric acid‑ethanol, and counterstained with eosin for cytoplasmic staining. After dehydration and clearing, the sections were mounted with neutral balsam. Histopathological morphology of each organ was observed and photographed under an optical microscope to evaluate potential tissue damage.

### 4.9 Establishment of In Vitro BBB Transwell Model and Assessment of Penetration Efficiency

The in vitro BBB Transwell model was set up as previously described. When the transendothelial electrical resistance (TEER) value reached 200–300 Ω cm [40], Cy5‐labelled DNA nanocircuit (400 nm) was added to the upper chamber (bEnd.3 cells) of the Transwell culture system (Corning) and co‐cultured with U87MG‐TR cells in the lower chamber. Samples were collected at 10, 30, 60, 120, and 240 min, and Cy5 fluorescence intensity in the lower chambers was visualized under a confocal microscope (Nikon).

### 4.10 Annexin V‐FITC/PI Flow Cytometry

Divide U87MG‐TR or NHA cells into 6 groups, and intervene with predetermined concentrations in each group. After processing, discard the culture medium, wash the cells twice with pre‐cooled PBS, digest and collect the cells with 0.25% trypsin, centrifuge at 1000 r/min for 5 min, and discard the supernatant. Add Annexin FITC binding solution to resuspend cells, adjust the cell concentration to 1 × 10^6^ cells/mL, take 100 µL of cell suspension, add 5 µL Annexin FITC and 5 µL propidium iodide (PI) staining solution, gently mix and incubate at room temperature in the dark for 15 min. After incubation, 400 µL of Annexin FITC binding solution was added, and the apoptosis rate was detected by flow cytometry within 1 h. FlowJo software was used to analyze the proportion of apoptotic cells.

### 4.11 Establishment of GBM Orthotopic Mouse Model

Luciferase‐lentivirus‐ transfected U87MG‐TR cells (5 × 10^5^ cells per mouse in 3 µL PBS) were injected into the brain of female BALB/c nude mice. The injection coordinates were set relative to the posterior lobe: 2.0 mm posterior, 2.0 mm lateral, and 3.0 mm ventral. Bioluminescence imaging was performed using the IVIS Spectrum system (PerkinElmer, USA) on days 5, 10, 15, and 20 post‐inoculation to monitor intracranial tumor growth. Body weight was recorded regularly, and survival analysis was conducted using the Kaplan–Meier method.

### 4.12 Anti‐GBM Efficiency of DNA Nanocircuit Combined With TMZ In Vivo

Ten days after the implantation of U87MG‐TR‐luc cells, GBM‐bearing mice were randomly allocated into 6 groups (n = 6 per group) and treated with indicated dosage combinations. From day 10 to 14 post‐inoculation, mice received daily tail vein injections of either the DNA nanocircuit or control nanostructure (400 nm, 200 µL per injection). Meanwhile, TMZ was administered intraperitoneally at 5 mg/kg. Body weights were recorded every four days.

Intracranial tumor progression was monitored on days 5, 10, 15, and 20 using bioluminescence imaging with the IVIS Spectrum system. At the end of the experiment, mice were sacrificed, and brain tissues were collected, fixed in 4% PFA, and embedded in paraffin for histological analysis, including H&E staining and IHC. For IHC, tissue sections underwent deparaffinization, rehydration, antigen retrieval, and blocking with 10% goat serum at room temperature for 1 h, followed by overnight incubation with primary antibodies (Ki67, PARP1) at 4°C. After PBS washing, HRP‐labeled secondary antibodies were applied, and signal detection was achieved with diaminobenzidine (DAB). Images were captured using a digital scanner (3DHISTECH, Hungary).

Additionally, protein levels of γH2Ax, Bax, Bcl‐2, and Caspase3 in tumor tissues were evaluated by western blotting, and densitometric analysis was performed with ImageJ. TUNEL staining was also conducted on paraffin sections to assess apoptosis, with nuclei counterstained using DAPI according to the manufacturer's instructions of the One‐Step TUNEL Apoptosis Detection Kit (KeyGEN BioTECH).

### 4.13 Statistical Analysis

Quantitative data were displayed as the mean ±SEM. Means were compared using the Student *t*‐test, and *p* values of less than 0.05 were considered statistically significant, where all significant values were indicated as follows: ns, no significant, ^*^ *p*<0.05, ^**^ *p*<0.01, ^***^ *p* < 0.001, ^****^ *p* < 0.0001.

## Author Contributions

**Jinjun He**: investigation, formal analysis. **Yueran Pan**: investigation, formal analysis. **Yan Zhao**: writing – original draft, funding acquisition, methodology. **Jinyue Fan**: formal analysis. **Haoxiang Chen**: data curation. **Wanxia Lu**: formal analysis. **Jong Seung Kim**: funding acquisition, conceptualization. **Min‐Goo Lee**: investigation, formal analysis. **Eunji Kim**: formal analysis. **Yuanbo Zhao**: data curation. **Xin Fu**: data curation. **Sungwook Jung**: data curation. **Yuling Xu**: conceptualization. **Chao Zhang**: conceptualization, writing – review and editing, supervision, funding acquisition. **Yufei Lan**: formal analysis, writing – original draft, investigation.

## Conflicts of Interest

The authors declare no conflicts of interest.

## Supporting information




**Supporting File**: advs77332‐sup‐0001‐SuppMat.docx.

## Data Availability

The data that support the findings of this study are available from the corresponding author upon reasonable request.
