# Supplementary material for: A Cascaded DNA Nanocircuit for Multi‐Signal‐Responsive Precision siRNA Delivery in Cancer Therapy
Source: Adv Sci (Weinh). 2026 Aug 24;13(63):e77332. doi: 10.1002/advs.77332 (PMC13502270; doi:10.1002/advs.77332)
Supplement: Supplementary file 1 [file ADVS-13-e77332-s001.docx]

Supporting Information

for

**A** **Cascaded DNA Nanocircuit for Multi-Signal-Responsive Precision siRNA Delivery in Cancer Therapy**

Yan Zhao^+^,^[a,b]^ Yufei Lan^+^,^[c]^ Min-Goo Lee^+^,^[d]^ Jinjun He,^[b]^ Yueran Pan,^[b]^ Eunji Kim,^[d]^ Xin Fu,^[c]^ Sungwook Jung,^[d]^ Wanxia Lu,^[c]^ Jinyue Fan,^[c]^ Haoxiang Chen,^[c]^ Yuanbo Zhao,^[c]^ Yuling Xu,*^[d]^ Jong Seung Kim,*^[d]^ and Chao Zhang*^[c]^

[a] School of Chemical Engineering and Technology, Sun Yat-sen University, Zhuhai 519082, China

[b] Institute of Biomedical Health Technology and Engineering, Shenzhen Bay Laboratory, Shenzhen 518132, China

[c] Department of Neurosurgery, Zhujiang Hospital, Southern Medical University, Guangzhou 510282, China

[d] Department of Chemistry, Korea University, Seoul 02841, Korea

[+] These authors contributed equally to this work.

*Correspondence: Chao Zhang [zc15996256330@smu.edu.cn](mailto:zc15996256330@smu.edu.cn); Jong Seung Kim [jongskim@korea.ac.kr](mailto:jongskim@korea.ac.kr); Yuling Xu [xuyuling@korea.ac.kr](mailto:xuyuling@korea.ac.kr)

# Supplementary tables and figures

**Figure S1.** DNA nanocircuit design for sequentially responsive process.

**Figure S2.** (a) AFM characterization of the DNA nanocircuit morphology under the four conditions. Average height (b) and width (c) statistics of the DNA nanocircuit, n = 20. All data are shown as mean ± SEM, ****P* < 0.001.

**Figure S3.** Native PAGE analysis verifying the two-step assembly of the DNA nanocircuit. Lane 1: core DNA nanocircuit assembled by annealing; Lane 2: ANG-conjugated DNA nanocircuit. Marker, DL2000.

**Figure S4.** Confocal images of U87MR-TR cells incubated with DNA nanocircuit. Green, cell membrane stained with Dio; blue, cell nucleus stained with DAPI; red, Cy5-labeled DNA nanocircuit.

**Figure S5.** Schematic drawing of two fluorophore-quencher pairs on DNA nanocircuit.

**Figure S6.** Quantitative fluorescence analysis of AND gate-1 under four input conditions. (a) A488 fluorescence intensity across the four input conditions (0,0), (1,0), (0,1), and (1,1). (b) AMCA fluorescence intensity across the four input conditions. Data are shown as mean ± SEM (n = 3). **P* < 0.05, ***P* < 0.01, ****P* < 0.001.

**Figure S7.** Fluorescence kinetics of the DNA nanocircuit in response to different inputs. (a) Fluorescence kinetics on channel AMCA. Gray, DNA nanocircuit without any input; purple, DNA nanocircuit in response to (1,1) input. (b) Fluorescence kinetics on channel A488. Gray, DNA nanocircuit without any input; blue, DNA nanocircuit in response to (1,1) input. Curves are shown as the mean ± SEM (n = 3).

**Figure S8.** PAGE analysis of siPARP1 release from the DNA nanocircuit under (0,1) and (1,1) conditions at an elevated GSH concentration of 10 mM. A647-labeled siPARP1 fluorescence remains associated with the nanocircuit in (0,1) condition, confirming that siRNA release requires concurrent AND gate-1 activation and GSH.

**Figure S9.** Native PAGE images (a) and quantification (b) of Cy7-labeled siPARP1 in DNA nanocircuit following incubation with 10% FBS over a series of time points. Data were presented as mean ± SEM (n = 3).

**Figure S10.** WB characterization (a) and quantitative analysis (b) of NCL expression on U87MG-TR cell surfaces under normal and starved conditions. The weak GAPDH signal confirms the high purity of our extraction with minimal cytosolic contamination. Data are shown as mean ± SEM (n = 3), ****P* < 0.001.

**Figure S11.** Raw images for Figure 3a.

**Figure S12.** Relative GSH content in BSO treated U87MG-TR cells. Data are shown as mean ± SEM (n = 5), *****P* < 0.0001.

**Figure S13.** Evaluation of DNA nanocircuit responsiveness in normal NHA cells. (a) Pseudocolor D/A images depicting the temporal response of AND gate-1 in NHA cells. Scale bar, 10 μm. (b) D/A ratio quantification from panel a, based on n = 10 fluorescent puncta per time point, shown as mean ± SEM. (c) Colocalization of the DNA nanocircuit (A488-labeled, green) and siPARP1 (A647-labeled, red) in AND gate-2. Line profiles show green and red fluorescence intensities along the dashed lines in the merged images. Scale bar, 10 μm. (d) Colocalization maps of the cells in panel c with the corresponding Pearson’s correlation coefficients, indicating siPARP1 sequestration without release.

**Figure S14.** Confocal fluorescence and brightfield images of cells in Figure 4a showing intracellular localization of the A488-labeled DNA nanocircuit (green). Nuclei are stained with DAPI (blue).

**Figure S15.** Serum biochemistry (ALT, AST, BUN, CR) and hematological parameters (RBC, WBC, HGB, PLT) in mice at Day 28 after administration of the DNA nanocircuit. Grey areas indicate standard reference ranges (n = 4). Data are shown as mean ± SEM.

**Figure S16.** Representative H&E staining of brain, heart, liver, spleen, lung, and kidney tissues from mice at Day 28 after DNA nanocircuit administration.

**Figure S17.** Cytokine profiling evaluating systemic immune responses at Days 7 and 28 after DNA nanocircuit administration. Data are shown as mean ± SEM (n = 6). ns, no significant difference.

**Figure S18.** *In vitro* characterization of DNA nanocircuit crossing the BBB Transwell model. (a) Construction of BBB Transwell model and the process of DNA nanocircuit crossing the upper chamber bEnd.3 cells into the lower chamber U87MG-TR cells. (b) The successful construction of the BBB model was demonstrated by the Transwell Chamber Transmembrane Resistance (TEER) experiment. Data are shown as mean ± SEM (n = 3).

**Figure S19.** Evaluation of apoptosis induced by the DNA nanocircuit in normal NHA cells.

**Figure S20.** CCK-8 assay for the cell viability of U87MG-TR cells after indicated treatments. Data are presented as mean ± SEM, ****P* < 0.001, *****P* < 0.0001.

**Figure S21.** Half-maximal inhibitory concentration (IC_50_) of TMZ on U87MG-TR cells when treated with indicated groups. Data are shown as mean ± SEM (n = 3).

**Figure S22.** *In vivo* distribution of Cy5 labeled-DNA nanocircuit with brain targeting peptide (ANG). (a) Representative bioluminescence and fluorescence images of the brain. (b) Representative immunofluorescent imaging of the GBM tissues. T: tumor; N: normal issue.

**Figure S23.** WB analysis of the molecular mechanism by which DNA nanocircuit promotes TMZ-rendered GBM therapy outcome. (a) WB images for protein expression. (b) Densitometric quantification of panel a (n = 3). Data in all panels are presented as mean ± SEM, **P* < 0.05, ***P* < 0.01, ****P* < 0.001.

**Figure S24.** TUNEL images detecting apoptosis of tumor cells in GBM tumor tissues after indicated treatments.

**Table S1.** DNA and peptide sequences used in this work.

| Name | Sequence (5’-3’) |
| --- | --- |
| 1 | CAGTTGACTGCTAGTACCTGAGCACTGAATGCGATGTAGAAGTAGCTCTGCTCCATC |
| 2 | AAAAAAAAAAAAAAAAAACGACTTGATGGAGCAGACCTATCGTCAC |
| 3 | AGGCAGATACGAAGAGCGCCACCACCTCGTAGATAGTTCTATAGGGGCGCTAGACAC |
| 4 | GCAGTCAACTG |
| 5 | AAAAAAAAAAAAAAAAAACGGTACGTGACGATAGGACACATCAGATGTCTTAGGAGAGGTCACAGTAACCTTCGACAATCT |
| 6 | AGATTGTCGAACGTATCTGCCT |
| 7 | GAACTATGACATCTGATGTGTGCTACTTGGCTGCC |
| 8 | CGCTCTTGGTTACTGTGACCTGTGCTCAGCCGCTG |
| 9 | GCATTCACTCCTAACTACGA |
| 10 | AAAAAAAAAAAAAAAAAATCTAGCGTGTCTAGCG |
| 11 | AAAAAAAAAAAAAAAAAATGTCCGTGCAACCGATCAATCC |
| 12 | GCCTAGCGATCCAATGGAACGACCGTATTGCTGAGGTGAGTGTATGTATCACTTGCACGGACA |
| 13 | CTGTACCGTTG |
| 14 | GGATTGATCGGATGCCAGACGCATCGGATTCGATGAGCCTACTCGACCAACTCAACG |
| 15 | AAAAAAAAAAAAAAAAAAGCGAGTGGCAATGTCCACCATTGGATCG |
| 16 | CAACGGTACAGGGCAGCCTCCAACCTTGTAACCAGCGGCATAACGCTGGACATTGCC |
| 17-imotif | CTATATCCCCTTACCCCTTACCCCTTACCCCCTATACTGGCATGTGATACATACACTGGTTGGA |
| 18-NCL aptamer | GGTGGTGGTGGTTGTGGTGGTGGTGGGGCTCATGCAATACGGTCGTTGCGTTAT |
| 19-siPARP1-sense | AAGAUAGAGCGUGAAGGCGAAdTdT(S-S)AACACCTCACGAATCC |
| 20 | AAAAAAAAAAAAAAAAAAGCACCACGTTGAGTTGG |
| siPARP1-  antisense | UUCGCCUUCACGCUCUAUCUUdTdT |
| 9-Cy3 | GCATTCACTCCTAACTACGA-Cy3 |
| 18-NCL aptamer-Cy5 | GGTGGTGGTGGTTGTGGT(Cy5)GGTGGTGGGGCTCATGCAATACGGTCGTTGCGTTAT |
| ANG-DNA | TTTTTTTTTTTTTTTTTT-Thr-Phe-Phe-Tyr-Gly-Gly-Ser-Arg-Gly-Lys-Arg-Asn-Asn-Phe-Lys-Thr-Glu-Glu-Tyr |
| 10-BHQ1 | AAAAAAAAAAAAAAAAAATCTAGCGTGTCTAGCG-BHQ1 |
| 17-imotif-A488 | CTATATCCCCTTACCCCTTACCCCTT(A488)ACCCCCTATACTGGCATGTGATACATACACTGGTTGGA |
| 9-AMCA | GCATTCACTCCTAACTACGA-AMCA |
| 18-NCL aptamer-Dabcyl | GGTGGTGGTGGTTGTGGT(Dabcyl)GGTGGTGGGGCTCATGCAATACGGTCGTTGCGTTAT |
| siPARP1-  antisense-A647 | A647-UUCGCCUUCACGCUCUAUCUUdTdT |
| 17-gate 1 closed | CCCCTATACTGGCATGTGATACATACACTGGTTGGA |
| 18-gate 1 closed | GGTGGTGGGGCTCATGCAATACGGTCGTTGCGTTAT |
| 17-gate 1 open | CTGGCATGTGATACATACACTGGTTGGA |
| 18-gate 1 open | GGCTCATGCAATACGGTCGTTGCGTTAT |
| 19 | AACACCTCACGAATCC |
| 19-siPARP1-sense-non res | AAGAUAGAGCGUGAAGGCGAAdTdTAACACCTCACGAATCC |

**Table S2.** DNA sequences required for the preparation of the DNA nanocircuit.

| DNA nanocircuit | DNA sequences |
| --- | --- |
| **Figure 2c** | 1, 2, 3, 4, 5, 6, 7, 8, 9-AMCA, 10-BHQ1, 11, 12, 13, 14, 15, 16, 17-imotif-A488, 18-NCL aptamer-Dabcyl, 19-siPARP1-sense, 20 |
| **Figure 2e** | 1, 2, 3, 4, 5, 6, 7, 8, 9, 1, 11, 12, 13, 14, 15, 16, 17-imotif, 18-NCL aptamer, 19-siPARP1-sense, 20, siPARP1-antisense-A647 |
| **Figure 3a** | 1, 2, 3, 4, 5, 6, 7, 8, 9-Cy3, 1, 11, 12, 13, 14, 15, 16, 17-imotif, 18-NCL aptamer-Cy5, 19-siPARP1-sense, 20 |
| **Figure 3b** | 1, 2, 3, 4, 5, 6, 7, 8, 9, 10, 11, 12, 13, 14, 15, 16, 17-imotif-A488, 18-NCL aptamer, 19-siPARP1-sense, 20, siPARP1-antisense-A647 |
| **Figure 4a AND-gate 1-deficient** | 1, 2, 3, 4, 5, 6, 7, 8, 9, 1, 11, 12, 13, 14, 15, 16, 17-gate 1 open-A488, 18-gate 1 open, 19-siPARP1-sense, 20, siPARP1-antisense-A647 |
| **Figure 4a cascaded AND gates** | 1, 2, 3, 4, 5, 6, 7, 8, 9, 1, 11, 12, 13, 14, 15, 16, 17-imotif-A488, 18-NCL aptamer, 19-siPARP1-sense, 20, siPARP1-antisense-A647 |
| **Figure 5 G3** | 1, 2, 3, 4, 5, 6, 7, 8, 9, 1, 11, 12, 13, 14, 15, 16, 17-imotif, 18-NCL aptamer, 19, 20 |
| **Figure 5 G4** | 1, 2, 3, 4, 5, 6, 7, 8, 9, 1, 11, 12, 13, 14, 15, 16, 17-gate 1 close, 18-gate 1 close, 19-siPARP1-sense-non res, 20, siPARP1-antisense |
| **Figure 5 G5** | 1, 2, 3, 4, 5, 6, 7, 8, 9, 1, 11, 12, 13, 14, 15, 16, 17-imotif, 18-NCL aptamer, 19-siPARP1-sense, 20, siPARP1-antisense |

**Table S3.** Antibodies used in this study.

| Antibodies | Manufacturer | Catalog | Dilution |
| --- | --- | --- | --- |
| Nucleolin | Abcam | ab22758 | WB: 1:1000  IF: 1:1000 |
| γH2Ax | Abcam | ab81299 | WB: 1:5000–1:10000  IF: 1:250 |
| Bax | Abcam | ab53154 | WB: 1:500–1:1000 |
| Bcl-2 | Abcam | ab194583 | WB: 1:500–1:2000 |
| Caspase3 | Abcam | ab32351 | WB: 1:5000 |
| ATPase | Abcam | ab76020 | WB: 1:100000 |
| β-actin | Proteintech | 20536-1-AP | WB: 1:1000–1:5000 |
| Ki67 | Abcam | ab16667 | IHC: 1:200 |
| Anti-rabbit IgG, HRP-linked Antibody | CST | #7074 | WB: 1:1000–1:3000 |
| Anti-rabbit IgG (H+L), F(ab’)_2_ Fragment (Alexa Fluor® 555 Conjugate) | CST | #4413 | IF: 1:500–1:2000 |
| DAPI | CST | #4083 | IF: 0.1–1 μg/mL |
| DyLight™ 488 Phalloidin | CST | #12935 | IF: 1:40 |

**Table S4.** Primers used in qRT-PCR.

| Genes | Forward primer (5’–3’) | Reverse primer (5’–3’) |
| --- | --- | --- |
| PARP1 | GCTCCTGAACAATGCAGACA | CATTGTGTGTGGCATGA |
| ACTB | TCGTGCGTGACATTAAGGAGAAGC | GGCGTACAGGTCTTTGCGGATG |

**[Uncropped raw images]**

**Figure 2e.**

**A647 channel**

**Gel-red channel**

**Figure 4d.**

**M.W.
(kDa)**

**PARP1 (113 kDa)**

**100**

**130**

**β-actin (42 kDa)**

**M.W.
(kDa)**

**40**

**55**

**M.W.
(kDa)**

**Figure 5c.**

**G2**

**G3**

**G4**

**G5**

**G1**

**PARP1 (113 kDa)**

**70**

**100**

**130**

**M.W.
(kDa)**

**55**

**β-actin (42 kDa)**

**40**

**35**

**Figure S6.**

**ATPase (100 kDa)**

**NCL (110 kDa)**

**M.W.
(kDa)**

**40**

**35**

**15**

**25**

**100**

**150**

**M.W.
(kDa)**

**40**

**35**

**15**

**100**

**150**

**25**

**M.W.
(kDa)**

**50**

**70**

**40**

**35**

**GAPDH (36 kDa)**

**Figure S12.**

**β-actin (42 kDa)**

**55**

**70**

**M.W.
(kDa)**

**G1**

**G2**

**G3**

**G4**

**G5**

**35**

**40**

**M.W.
(kDa)**

**55**

**40**

**Caspase3 (32 kDa)**

**)**

**35**

**25**

**M.W.
(kDa)**

**35**

**Bcl-2 (26 kDa)**

**25**

**25**

**M.W.
(kDa)**

**M.W.
(kDa)**

**Bax (21 kDa)**

**)**

**M.W.
(kDa)**

**35**

**γH2Ax (16 kDa)**

**25**

**G1**

**G2**

**G3**

**G4**

**G5**

**G6**

**M.W.
(kDa)**

**Figure S13.**

**55**

**40**

**β-actin (42 kDa)**

**35**

**M.W.
(kDa)**

**55**

**40**

**Caspase3 (32 kDa)**

**35**

**25**

**M.W.
(kDa)**

**35**

**25**

**Bcl-2 (26 kDa)**

**M.W.
(kDa)**

**25**

**Bax (21 kDa)**

**M.W.
(kDa)**

**25**

**15**

**γH2Ax (16 kDa)**
